# The impact of COVID-19 pandemic on hand hygiene adherence among pediatric physicians, in Saudi Arabia

**DOI:** 10.1016/j.amsu.2022.104518

**Published:** 2022-09-06

**Authors:** Abdullah AlGhobaishi, Haifa Alzabin, Asma Alhazmi, Ahmed Hafez Mousa, Hatem AlThagafi, Mohammed Alghamdi, Adeeb Khawaji, Eyad Albenayan, Roaa Zailaie, Rana Hassan Almaghrabi, Mohammed A. Garout

**Affiliations:** a Department of Pediatrics, King Fahad Armed Forces Hospital, Jeddah, Saudi Arabia; b Emergency Department, King Fahad General Hospital, Jeddah, Saudi Arabia; c Department of Infectious Diseases, King Fahad Armed Forces, Jeddah, Saudi Arabia; d Department of Pediatrics Infectious Disease, King Fahad Armed Forces, Jeddah, Saudi Arabia; e Department of Pediatrics, Prince Sultan Military Medical City, Riyadh, Saudi Arabia; f Faculty of Medicine, Department of Community Medicine, Umm Al Qura University, Makkah, Saudi Arabia; g Central Board for Accreditation of Healthcare Institutions (CBAHI) Control Leader, Saudi Arabia; h College of Medicine and Surgery, Batterjee Medical College, Jeddah, Saudi Arabia; i Children's Health Center, Department of Pediatrics, International Medical Center, Jeddah, Saudi Arabia

**Keywords:** COVID-19, Knowledge, Awareness, Adherence, Pediatrician, Hand hygiene practice

## Abstract

**Introduction:**

The hand hygiene practice (HHP) is the most effective and simplest preventive measure **to** reduce the risk of infection. HHP is more relevant among pediatric physicians in the context of the COVID-19 pandemic since, children are more vulnerable to infection. Therefore, assessment of the COVID-19 impact on HHP could be useful in minimizing lethal virus transmission from pediatric physicians to patients and vice versa.

**Method:**

The present cross-sectional, electronically self-administered supplement based survey study was conducted among different professional levels of pediatric physicians involving consultants, specialists, and residents. The supplement includes information related to demography, knowledge, awareness, preventive measures, demonstration and practice of HHP. The information was collected and summarized on a Microsoft excel sheet before being imported to SPSS for statistical analysis.

**Results:**

Of the total (N = 404) pediatric physicians, 56.68% male, 43.06% belongs to 25–35 years, 42.32% were consultants, 98.01% respondents were familiar with five moments of HHP. Further, HHP immediately before touching patients (99.26%), clean/aseptic procedure (95.04%), after body fluid exposure (72.28%), after touching patients **(**98.01%), after touching surrounding of patients (74.75%) may prevent germ transmission to patients whereas HHP after touching patients (98.27%), before clean/aseptic procedure (67.57%), after exposure to immediate surroundings of patients (97.02%) may prevent germ transmission to pediatric physicians. Rubbing hands is preferred before palpation of abdomen (74.25%), before giving injection (56.68%), after removing gloves (61.88%), after making a patient's bed (47.80%), while washing of hands preferred after emptying bedpan (67.82%) and after visible exposure to blood (84.40%), 92.57% believed gloves can't replace HHP, posters display at point of care as reminders (95.30%), received frequent HHP education (82.92%), 50.49% do not need HHP reminder, 51.73% preferred alcohol based sanitizer, 53.46% facilitate daily morning huddle, HHP >10 times per day before COVID-19 (24.62%) while in COIVID-19 (56.44%). HPP is the most effective way to prevent the spread (98.01%) of microbes because it kills germs (90.35%), health care associated infections is the major (38.06%) cause of germ transmission, 86.88% will be remains committed to HHP even after pandemic. In comparison to residents and specialists, consultants gave more importance (p = 0.02) to HHP and were more adherent during (p = 0.007) and even after (p = 0.001) COVID-19 pandemic.

**Conclusion:**

Assessing knowledge of pediatric physician, awareness, and adherence to hand hygiene measures could be helpful to reduce the contact transmission of lethal viruses to patients and vice versa. Further increase in the awareness, knowledge and education of HHP are required in order to maximize its utilization.

## Highlights

• The hand hygiene practice (HHP) is the most effective and simplest preventive measure **to** reduce the risk of infection.
• Of the total (N = 404) pediatric physicians, 56.68% male, 43.06% belongs to 25–35 years, 42.32% were consultants, 98.01% respondents were familiar with five moments of HHP.
• Assessing knowledge of pediatric physician, awareness, and adherence to hand hygiene measures could be helpful to reduce the contact transmission of lethal viruses to patients and vice versa.

## 1 Introduction

**Fig. 1:**
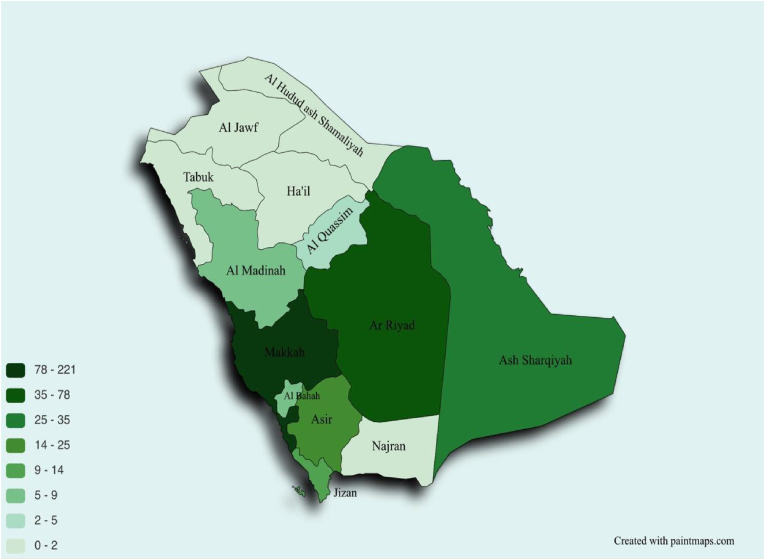
Showed location of the respondent residence.

**Fig. 2:**
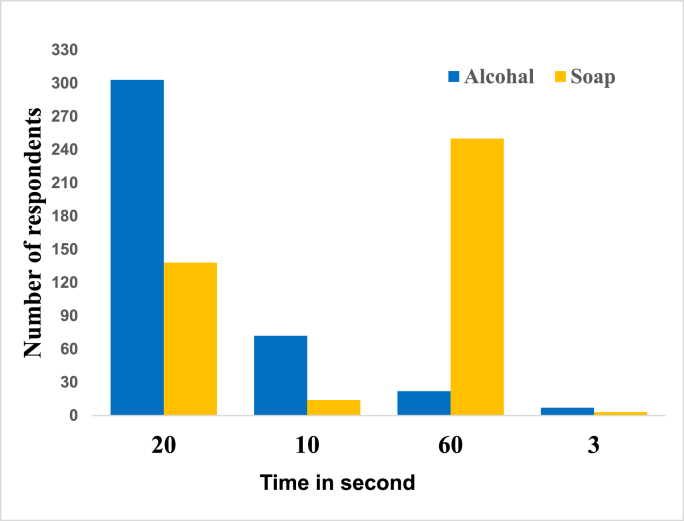
Showed the comparison of attitudes of respondents toward the use of alcohol and soap at various time intervals, N = 404 where N = total number of respondents.

**Fig. 3:**
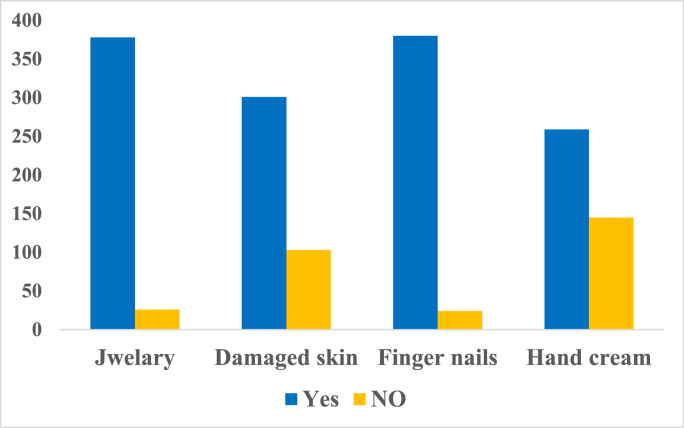
Showed the comparison of respondents' perceptions of associated risk factors for SARS CoV-2 transmission, N = 404 where N = total number of respondents.

**Fig. 4:**
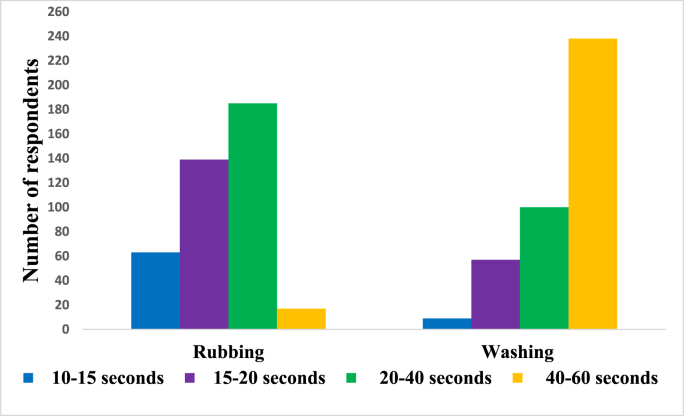
Showed the comparison of the duration of hand rubbing and washing at various time interval, N = 400 where N = total number of respondents.

In December 2019, an outbreak of coronavirus disease (COVID-19) caused by Severe Acute Respiratory Syndrome Coronavirus 2 (SARSCoV-2) was detected in Wuhan, China and then became unprecedented global health crisis. Healthcare workers (HCWs) are the frontline fighters during COVID-19 pandemic, so they are more likely to get infected with SARS-CoV-2 which appears to be transmitted primarily through respiratory droplets, from face-to-face contact and, to a lesser extent, through contaminated surfaces. Our hands are the most critical body part for transferring pathogens [[3], [8]]. Several methods have been suggested to relower the rate of SARSCoV-2 transmission such as hand hygiene practice (HHP), social distancing, the use of gloves etc [6]. The hand hygiene practice is the most effective, simplest preventive measure that can decrease the proliferation of microorganisms as a results reducing risk of infection and overall healthcare costs, length of stays, and reimbursement [35]. The WHO and the CDC have repeatedly emphasize the importance of HHP for reducing the transmission rate of COVID-19 infections worldwide **(**18**).** It can be performed by washing hands with an alcohol based hand rub or wash hands with either plain or antimicrobial soap or water [[13], [14]]. In today's world, social media has made HHP-related material more accessible and pervasive resulting in increased awareness and importance of HHP in the daily lives of general population, politicians, public figures, and many others [[1], [2]]. Continuous efforts are being made to identify effective and sustainable ways to overcome the lack of compliance with hand hygiene. One of such efforts is the introduction of an evidence-based concept of “my five moments for hand hygiene” by World Health Organization [7]. Ataee et al., 2017 reported growing awareness in HCWs on the importance of hand washing causes a reduction of about 30% in the transmission of infectious agents [5]. Despite repeated calls to increase healthcare workers' awareness of, access to, and adherence to hand hygiene and mask wearing guidelines, HCW adherence remains low in many places. In USA alone, only 50% hospitals are stick with standard hand hygiene [[24], [25]]. Moore et al., 2021 reported a dramatic increase in COVID-19 morbidity with a drop in hand hygiene compliance [27]. Among all healthcare workers, pediatric physicians have a heightened responsibility to adhere with HHP in the context of the COVID-19 pandemic [[16], [17]] since, children, are often described to have an ‘immature’ immune system and, for infections with respiratory tract viruses such as SARSCoV-2 either by contact or droplet transmission [[18], [39]]. Ministry of health in Saudi Arabia did a significant effort in COVID-19 pandemic reminding and aware healthcare workers and general population to adhere on hand hygiene, following the WHO guidelines. The government has closely monitoring the situation and developing country-specific measures that are in line with the WHO guidelines in dealing with the outbreak in kingdom of Saudi Arabia. There are many infection control strategies which have been used to reduce risk of SARS-CoV-2 in HCPs. The impact of COVID-19 on HHP in healthcare settings is currently unclear due to a lack of proper evidences. Hence, the current study aims to conduct a cross-sectional study using electronic survey to assess knowledge, attitudes, and awareness of HHP and also level of adherence of pediatric physicians to the preventive measures against COVID-19 in Saudi Arabia. This work has been done in line with the STROCSS criteria [41] (see Table 1, Table 2, Fig. 1, Fig. 2, Fig. 3, Fig. 4).

**Table 1:** Showed demographical characteristics, knowledge, awareness, demonstration and practice of HHP practice among pediatricians.

Demographic characteristics	Number (N = 404)	Percentage (%)
**Gender**		
Male	229	56.68
Female	175	43.32
**Age (Years)**		
25–35	174	43.06
35–45	122	30.20
45–55	89	22.02
>60	19	4.70
**Providences/location**		
South of Saudi Arabia	34	8.41
Central Saudi Arabia	98	24.25
East of Saudi Arabia	35	8.66
West of Saudi Arabia	230	56.93
North of Saudi Arabia	07	1.73
**Professional ranks**		
Consultant	171	42.32
Residents	127	31.44
Specialist	106	26.24
**Departments**		
General pediatrics	166	41.08
Critical department	150	37.13
Pediatric subspecialty	88	21.78
**Knowledge and awareness**		
**Do you know the 5 moments for HHP by the World Health Organization?**		
Yes	396	98.01
No	08	1.99
**Do you think HHP before touching a patient may prevents transmission of germs to the patient?**		
Yes	401	99.26
No	03	0.74
**Do you think HHP immediately after a risk of body fluid exposure prevents transmission of germs to the patient?**		
Yes	292	72.28
No	112	27.72
**Do you think HHP after touching a patient may prevents transmission of germs to the patient?**		
Yes	396	98.01
No	08	1.99
**Do you think HHP after exposure to the immediate surroundings of a patient prevents transmission of germs to the patient?**		
Yes	302	74.75
No	102	25.25
**Do you think HHP immediately before a clean/aseptic procedure prevents transmission of germs to the patient?**		
Yes	384	95.04
No	20	4.95
**Do you think that HHP after touching a patient prevents transmission of germs to the health-care worker?**		
Yes	397	98.27
No	07	1.73
**Do you think that HHP immediately before a clean/aseptic procedure prevents transmission of germs to the health-care worker?**		
Yes	273	67.57
No	131	32.43
**Do you think that HHP after exposure to the immediate surroundings of a patient prevents transmission of germs to the health-care worker?**		
Yes	392	97.02
No	12	2.98
**What is the most frequent source of germs responsible for health care associated infections?**		
I. Close contact with other health care workers	32	7.92
II. Germs already present on or within the patient	35	8.66
III. The hospital environment	157	38.86
IV. The hospital's water system	06	1.49
V. The hospital air	07	1.73
I + II	07	1.73
II + III	32	7.92
I + II|+III	36	8.91
V + I + II	25	6.18
III + II + I	28	6.93
III + II + I + IV + V	39	9.65
**Change in percentage perception about importance of HHP since the start of the pandemic**		
100%	187	46.28
75%	102	25.24
50%	64	15.85
25%	51	12.63
**Before palpation of the abdomen which type of HHP method is required**		
Rubbing	300	74.25
Washing	101	25
None	03	0.74
**Before giving an injection which type of HHP method is required**		
Rubbing	229	56.68
Washing	171	42.33
None	04	0.90
**After removing examination gloves which type of HHP method is required**		
Rubbing	250	61.88
Washing	144	35.64
None	10	2.47
**After emptying a bedpan which type of HHP method is required**		
Rubbing	115	28.47
Washing	274	67.82
None	15	3.71
**After making a patient's bed which type of HHP method is required**		
Rubbing	193	47.80
Washing	191	47.28
None	20	4.92
**After visible exposure to blood which type of HHP method is required**		
Rubbing	52	12.87
Washing	341	84.40
None	11	2.72
**The use of gloves replace the need for hand hygiene?**		
Yes	30	7.42
No	374	92.57
**Does COVID-19 pandemic affected your knowledge about the 5 moments of hand hygiene?**		
Yes	265	65.60
No	139	34.40
**HHP posters are displayed at point of care as reminders?**		
Yes	385	95.30
No	19	4.70
**Do you receive frequent education on hand hygiene?**		
Yes	335	82.92
No	69	17.07
**Do you need to remember or to be reminded to do hand hygiene?**		
Yes	200	49.50
No	204	50.49
**Do you think that using alcohol based hand sanitizer is less time consuming and efficient rather than hand washing with water and soap?**		
Yes	209	51.73
No	142	35.14
I don't know	53	13.12
**Demonstration and practice**		
**Do the institution facilitate daily morning huddle about HHP occurs frequently?**		
Yes	216	53.46
No	188	46.53
**Before COVID-19 pandemic, how many times you were washing or hand rub your hands per day?**		
2-4 times	95	23.51
4-6 times	82	20.29
6-8 times	60	14.85
8-10 times	69	17.07
>10 times	98	24.26
**Since the start of the pandemic, how many times you are washing your hands per day?**		
2-4 times	11	2.72
4-6 times	39	9.66
6-8 times	60	14.85
8-10 times	66	16.33
>10 times	228	56.44
**What's your about HHP during this pandemic is important and best way to prevent spreading COVID-19 infection?**		
**Percentage adherent to HHP after COVID- 19 pandemic**		
100%	226	55.94
75%	123	30.45
50%	36	8.91
25%	19	4.70
Yes	396	98.01
No	8	1.99
**Why do you think HHP during this pandemic is important and best way to prevent spreading COVID-19 infection?**		
It will kill the germs	365	90.35
It will not kill the germs	39	9.65
**COVID-19 pandemic lasts longer time than the expected, after one year, did you become less strict about hand hygiene?**		
Yes	131	32.42
No	273	67.57
**What's obstacles you are facing regarding hand hygiene?**		
Forgetting	59	14.60
Forgetting, unavailability	15	3.72
Hand dryness, forgetting	125	30.94
Hand dryness, forgetting, unavailability	8	1.98
Hand dryness, time consuming, forgetting	36	8.91
Hand dryness, unavailability, strong smell of the products	31	7.67
None	116	28.71
What's adherence percentage difference at the start of COVID outbreak and now		
100%	84	20.79
75%	112	27.72
50%	73	18.06
25%	135	33.41

**Have you been infected by COVID-19?**		
Yes	98	24.25
No	305	75.50
Not sure	1	0.25
**If infected, how?**		
Close contact to COVID positive patient	93	94.90
Don't know	05	5.10
**Do you feel you are exhausted by all of the personal protective equipment (PPE) to prevent COVID outbreak?**		
Somehow	147	36.38
Yes	125	30.94
No	132	32.67
**If the outbreak is over, you will be committed to hand hygiene?**		
Yes	351	86.88
No	14	3.46
Probably	39	9.65

HHP= Hand hygiene practice, percentage are shown in parenthesis, N = Number of participants.

**Table 2:** Shows the importance of hand hygiene, its adherence and changes in hand hygiene practice after COVID-19 pandemic The Chi square test was performed to examine the importance of hand hygiene, its adherence, and changes in hand hygiene following the COVID-19 pandemic among the various levels of professionals. HHP = hand hygiene practice, percentage are shown in parenthesis, and bold values were significant at *p* < 0.05. N= Number of respondents.

Professional's ranks	Importance of HHP (%)				*p*-values
N = 404	100%	75%	50%	25%	
Consultant (170)	81 (20.04)	38 (9.40)	27 (6.68)	24 (5.94)	
Resident (129)	54 (13.36)	47 (11.63)	17 (4.20)	11 (2.72)	
Specialist (105)	50 (12.37)	18 (4.45)	21 (5.19)	16 (3.96)	**0.02**
**Adherent to HHP after COVID- 19 pandemic (%)**					
Consultant (170)	106 (26.23)	41 (10.14)	12 (2.97)	11 (2.72)	
Resident (129)	57 (14.10)	55 (13.61)	14 (3.46)	03 (0.74)	
Specialist (105)	59 (14.60)	29 (7.17)	12 (2.97)	05 (1.23)	**0.007**
**Post COVID-19 changes in HHP adherence (%)**					
Consultant (170)	46 (8.29)	41 (10.14)	21 (5.19)	62 (15.34)	
Resident (129)	19 (4.70)	38 (9.40)	31 (7.67)	41 (10.14)	
Specialist (105)	61 (15.09)	27 (6.69)	11 (2.71)	05 (1.23)	**0.001**

## 2 Methods

The current multi-center, cross-sectional supplement-based survey study was conducted among pediatrics physician during the period of 17 September 2021 to 28 October 2021 to assess the impact of the COVID-19 pandemic on HHP in the Kingdom of Saudi Arabia. An electronically self-administered supplement was designed and modified based on a previously published study [15,29,36]. The supplement was revised by pediatricians and infection management experts and was adopted in both Arabic and English languages. The institutional ethical clearance was obtained from the institutional review board of King Fahd Armed Forces Hospital, Jeddah, Saudi Arabia before conducting the research study. Prior to data collection, study participants gave their informed, explicit consent to have their data published. We included only those HCPs who are registered in the human resources department and work as pediatricians in various hospitals and accepted the invitation; excluded all those who refused to complete the online survey, practicing other than pediatric department, and non-healthcare workers. The participants received the supplement by email, WhatsApp, and Google forms. The supplement included thirty blind questions comprised three parts: the first part covered socio-demographic variables such as age, gender, residence location, professional ranks, and departments. Further, gender was coded as one for men, and zero for women. The age variable was divided into categories into 25–35, 35–45, 45–55 and > 60. Professional rank was categorized and coded consultant as one, resident as two and specialist three, general pediatrics as 1, critical department as two and pediatric subspecialty as three. Residence location were coded **s**outh of Saudi Arabia as one, central Saudi Arabia as two, east of Saudi Arabia as three, west of Saudi Arabia as four and north of Saudi Arabia as four. The second part included questions based on knowledge, awareness and preventive measures. Five questions were developed based on WHO's ‘My five moments for hand hygiene,’ which include HHP before patient contact, before clean/aseptic procedures, after the risk of body fluids exposure, after patient contact, and after contact with patient surroundings to assess self-reported HHP or compliance. Respondents were asked to choose whether they agreed (yes) or disagreed (no) with each of the five questions [9]. The third part comprised question related demonstration and practices; the respondents reported the number of times they wash or rub their hands per day in normal days and during the COVID-19 pandemic, whether HHP is the best way to prevent spreading COVID-19 infection, will the responders become less strict if pandemic run for longer time, have responders been affected by COVID-19, will the responder perform HHP (Y/N) after outbreak, minimal time duration for alcohol, soap and water based HHP etc. The data were collected by five trained health professionals. They were trained on the objectives of the study and the contents of the data collection checklist. To avoid selection bias, data collection was closely supervised by the principal investigators. The data was manually verified before being transferred to a computer and entered into a Microsoft excel spreadsheet. All the possible statistical analysis was conducted to find out the impact of study.

### 2.1 Statistical analysis

The statistical analysis was carried out by using SSPS software, windows version 21.0 (Chicago,IL, USA).The data was presented using frequency, percentage, arithmetic mean, and standard deviation.

Chi-squared test/Fisher's exact test was used for the analysis of the impact of professional ranks on the importance, and adherence to HHP. The *p* < 0.05 were considered as significant values.

## 3 Results

Of the total (N = 404) pediatric physicians invited to fill the questionnaire consisting 229 males (56.68%) and 175 females (43.32%). Most of the participants’ ages were under the age group of 25–35 years (43.06%). Majority of participants were consultants 171 (42.32%), followed by residents 127 (31.44%) and specialist 106 (26.24%). The most of respondents worked in general pediatrics department 166 (41.08%) followed by critical department 150 (37.13%) and another pediatric subspecialty 88 (21.78%) are shown in table-1. The provincial distribution data of respondents revealed that most of the respondents were from Riyadh followed by Makkah province (Figure-1). In comparison to residents and specialists, consultants gave more significance (p = 0.02) to HHP and were more adherent to HHP during (p = 0.007) and even after (p = 0.001) the pandemic (Table-2). A comparison of respondents' attitudes toward the use of alcohol and soap at various time intervals were presented in figure-2. Alcohol based HHP (303/404, 75%) at 20 s and soap at 60 s (250/404, 61.88%) were the most preferred (Figure-2). When comparison was made to find out the associative risk factors for the increased risk of COVID-19, we found Jewelry and finger nails ranked top (Figure-3). In Figure-4, demonstration of the duration of hand rubbing and washing in order to prevent viral transmission have shown that 20–40 s for hand rubbing and 40–60 s for hand washing were preferred.

When data were analyzed for the five moments of HHP, we found 396 (98.01%) respondents were familiar with my five moments of HHP, 265 (65.60%) thought that hand hygiene affected during this pandemic, unfortunately, 98 (24.25%) had been infected with covid-19.

HHP immediately before touching patients (99.26%), clean/aseptic procedure (95.04%), after body fluid exposure (72.28%), after touching patients (98.01%), after touching surrounding of patients (74.75%) may prevent germ transmission to patients whereas HHP after touching patients (98.27%) before clean/aseptic procedure (67.57%), after exposure to immediate surroundings of patients (97.02%) may prevent germ transmission to pediatric physicians.

Rubbing hands is preferred before palpation of abdomen 300 (74.25%), before giving injection 229 (56.68%), after removing gloves 250 (61.88%), after making a patient's bed 193 (47.80%), while washing hands preferred after emptying bedpan 274 (67.82%) and after visible exposure to blood 341 (84.40%), 374 (92.57%) believed gloves can't replace HHP, posters display at point of care as reminders (95.30%), 332 (82.92%) received frequent HHP education, 204 (50.49%) do not need reminder for HHP, 209 (51.73%) preferred alcohol based sanitizer, 216 (53.46%) facilitate daily morning huddle, before COVID-19 only 98 (24.26%) adopting >10 times HHP while 228 (56.44%) since the start of the pandemic, 396 (98.01%) believe that HHP is the best to prevent spreading of COVID-19 infection, 365 (90.35%) HHP kills germ, 273 (67.57%) would be last longer than expected, 125 (30.94%) hand dryness forgetting is the major obstacle in HHP, 24.25% infected by COVID-19 of which 94.89% because of close contact, 147 (36.38%) may prevent COVID-19 outbreak, 351 (86.88%) will be committed to HHP even after this pandemic (Table-1).

## 4 Discussion

The unprecedented COVID-19 outbreak and its high transmission rate become a major threat, globally. Despite the efforts of the entire world to comprehend COVID-19, understanding of this novel virus remains limited, and there is no medication therapy to cure COVID-19. Preventing spread is extremely important to reduce the overall burden of the disease and to remain safe. Healthcare professional are front warier therefore, they are at high risk of exposure and had the likelihood of acquiring this disease more. Currently, WHO recommends physical distances, appropriate use of all PPE, and HH practices to reduce the spread [12,23]. It is recommended to HCPs to practice prevention measures as the best solution. Hence, effective prevention can be achieved by addressing the problems by improving knowledge, attitude, and practices towards COVID-19.

Our study demonstrates male respondents were higher than female and more than half of respondent's age was lying between 25 and 35 years, consultant and general pediatric physicians were rank top among the responders. The higher response rate from high-ranking experts of pediatrics departments may be useful in evaluating pediatrics physician approach for safe clinical practices during COVID-19 in Saudi Arabia. Our study have shown consultants prioritize the hand hygiene (*p* = 0.02), adherent to HHP during (*p* = 0.007) and even after COVID-19 pandemic (*p* = 0.001) significantly as compared to residents and specialist (Table-2). Respondents from major cities, like Riyadh, indicating HHP is more important to them. (Figure-1). It has been suggested that knowledge of good HHP and compliance in hand hygiene as per WHO guidelines is essential for lowering COVID-19 infection [30]. In order to ensure good hand hygiene to prevent infection, WHO launched a well-defined clear approach of my five moments of hand hygiene to assess the level of knowledge and awareness. The current study revealed that more than 98% of respondents knew the five moments of hand hygiene, of which 99.26% believed that HHP before touching patients, 95.04% clean/aseptic procedure. 98.01% HHP after touching patients, 74.75% after touching immediate surroundings of patients may prevent germ transmission to patients. The proportion of respondents who believe that HHP immediately after a risk of body fluid exposure prevents germ transmission to patients is 72.28%, which is lower than the Indian study of 83.35%. Similarly, 98.27% of respondents believes HHP after touching a patients, 67.57% before clean/aseptic procedure, and 97.02% after exposure to immediate surroundings of patients may prevent germ transmission to pediatric physicians. Similar opinions were expressed by 96.9% of the Indian that HHP after touching a patients may prevent germ transmission to HCW. Our findings revealed the key issues on the importance of HHP and transmission of germs from pediatrics physician to patient and vice versa. There is sufficient scientific data to support the idea that appropriate hand cleanliness can considerably lower the risk of infection transmission from pediatric healthcare providers to patients [6,22]. The hospital environment is one of the major causes of HCAIs and considered as major safety concern for both health care providers and patients. In our study, to lower HCAIs risk, rubbing is preferred by 74.25% of respondents before palpation of abdomen, 56.68% before giving injection, 61.88% after removing examination gloves, 47.80% after making a patients bed pan whereas washing is preferred by 67.82% after emptying bed pan, 84.40% after visible exposure to blood, 92.57% believes that gloves can't replace HHP. The high percentage of compliance with HHP in our study is in line with Wong et al., 2021 who suggested, it could be effective in improving clinical environments, protecting patients and HCWs from COVID-19 infection. Respondents in our study also believe that wearing artificial jewelry, fingernails, damaged skin and hand cream could be sources of germ transmission in hospital settings (Figure-3). Our findings are in agreement with those of Suen et al., 2020 from Hong Kong and Maheshwari et al., 2014 from Bhopal. After the outbreak, perceptions of pediatric physicians for the importance of HHP changed significantly, according to our findings. 46.28% respondent thought the importance of HHP has increased by hundred percent since the start of the pandemic. Furthermore, following the COVID-19 pandemic, 55.94% of responders were found to be 100% HHP adherent. However, between the start of the COVID outbreak and now, only 33.41% of responders showed a change of 25% in HPP adherence at the start of COVID outbreak and now.

Alcohol based hand rub is one of the most effective methods to prevent infections due to its lower coast, easy availability and simple way to use. It acts by causing viruses to swell and burst by denaturing proteins and inactivated membranes (Golin et al., 2020). In our study, the respondents preferred the application of alcohol for 20 s and soap for 60 s. Furthermore, respondents believed that hand rubbing for 20–40 s and hand washing for 40–60 s may prevent viral transmission (Figure-4). It has been recommended that regular and thorough hand washing with alcohol for 20 s and soap for at least 40 s can remove viruses from the hands. Similar findings were also reported in Indian population, 51.73% agreed that using alcohol based hand sanitizer is less time consuming and efficient than hand washing with water and soap. According to our findings, more than half of respondents believes frequent education on HHP, poster exhibition could serve as HHP may improve the knowledge, awareness and reminders, more than half believe that ABHS is less time consuming and efficient than washing hand with water and soap. Hand washing with soap and water or using an ABHS is a simple yet efficient strategy to reduce the transmission of COVID-19 in hospital settings [[20], [24],^34^]. The WHO recommends ABHS over soap and water hand washing in most clinical situations unless the hands are visibly contaminated. It should be since ABHS is time and cost effective, readily available at the point of service, has enhanced skin tolerability, and covers a broad microbiological spectrum [[19], [28]]. To eliminate the COVID-19 virus, the ABHS must include at least 60% ethanol or 70% isopropanol [10,21,31].

When the data was evaluated to compare hand hygiene compliance before and after the pandemic, we observed hand washing or rubbing >10 times increased from 24.26% to 56.44%. Our data showed, the increase in the level of awareness and importance of hand hygiene among respondents.

However, we noticed that after one year of the pandemic, 32.42% of the pediatrics physicians became less strict about hand hygiene. One likely cause is hand dryness and forgetfulness, which prevent proper HHP in our study. Other than this, hand dryness and eczema can also influence adherence of hand hygiene. American contact dermatitis society published a recommendations to prevent hand dryness especially among health care workers during COVID-19 pandemic suggesting using less irritating agents such as alcohol based rub instead of frequent hand washing [[4], [32], [33], [37]].

Similar to this, a study conducted in nine hospitals of United States to observe the impact of COVID-19 on hand hygiene using electronic monitoring, results showed significant increase in awareness and adherence at the start of the pandemic. In a reassurance responses, we found 86.88% of the respondent will be committed to hand hygiene even after the pandemic. In order to review the study group perspective regarding what do they think for the most frequent source of germs transmission, 38.86% stated that it is the hospital environment such as surfaces, doors handles. However, in an Indian study, 34.05% of healthcare workers believe it's the germs existing on or inside the patient. Several other studies have shown that HCWs compliance with hand hygiene recommendations is generally poor, and evidence regarding the proper usage of PPE is few and underwhelming [26]. The fact that SARS-Cov2 is transmitted via aerosol is a major factor to consider [[38], [40]]. The outcome of the present provided substantial evidence to facilitate the development and implementation of an action plan, designing the internal guideline and policies as well as national policies for the requirement of hand hygiene among pediatric physician. Our findings further highlight the importance of training to improve HHP compliance among pediatrics physician. In addition, the findings of our research can be utilized by the scientific community for future investigation.

## 5 Conclusion

Assessing knowledge of pediatric physician, awareness, and adherence to hand hygiene measures may help to reduce the contact transmission of lethal viruses to infants, children and HCW. Our data evidenced that proper hand hygiene implementation can significantly reduce risk of germ transmission from pediatric healthcare practitioners to patients or vice versa. Our study has certain limitations in terms of the self-reporting of HHP performance and volunteer bias. To support the current findings, a larger cross-sectional study with a bigger sample size is required to generalize the findings of the study.

## Ethical approval

Ethical approval has been given by the Institutional Review Board (IRB) of our institution.

## Source of funding

NO FUNDING FOR THIS RESEARCH.

## Author contributions

Drafting of the manuscript: All authors contributed to the drafting of this manuscript,

Critical revision of the manuscript for important intellectual content: All authors contributed to the critical revision of the manuscript.

## Trail registry number

NONE.

## Guarantor

Abdullah AlGhobaishi, corresponding author of the manuscript, accept full responsibility for the work and the conduct of the study, had access to the data, and controlled the decision to publish.

## Declaration of competing interest

NO CONFLICTS OF INTREST.
